# Maternal obesity and severe pre-eclampsia among immigrant women: a mediation analysis

**DOI:** 10.1038/s41598-020-62032-9

**Published:** 2020-03-23

**Authors:** Ayesha Siddiqui, Catherine Deneux-Tharaux, Dominique Luton, Thomas Schmitz, Laurent Mandelbrot, Candice Estellat, Elizabeth A. Howell, Babak Khoshnood, Nathalie Bertille, Elie Azria

**Affiliations:** 1 INSERM U1153 Obstetrical, Perinatal and Pediatric Epidemiology Research Team (EPOPé), Center for Epidemiology and Statistics Sorbonne Paris Cité, Risks in Pregnancy DHU, Paris Descartes University, Paris, France; 2 Department of Family Medicine and Community Health, Icahn School of Medicine at Mount Sinai, New York, NY USA; 3 Department of Obstetrics and Gynecology, Beaujon-Bichat Hospital, University Hospital Department (DHU) Risks in Pregnancy, Paris Diderot University, Paris, France; 4 Department of Obstetrics and Gynecology, Robert Debré Hospital, Paris Diderot University, Paris, France; 5 Department of Obstetrics and Gynecology, Louis Mourier Hospital, University Hospital Department (DHU) Risks in Pregnancy, Paris Diderot University, Colombes, France; 6 Pitié-Salpêtrière Hospital – Charles Foix; Department of Biostatistics, Public Health and Medical Information, Clinical Research Unit, Pharmacoepidemiology Center (Céphépi), Sorbonne University; INSERM UMR-S 1136 - Pierre Louis Institute of Epidemiology and Public Health, Paris, France; 7 Department of Population Health Science and Policy, Icahn School of Medicine at Mount Sinai, New York, NY USA; 8 Department of Obstetrics, Gynecology, and Reproductive Science, Icahn School of Medicine at Mount Sinai, New York, NY USA; 9 Blavatnik Family Women’s Health Research Institute, Icahn School of Medicine at Mount Sinai, New York, NY USA; 10 Maternity Unit, Notre Dame de Bon Secours -Paris Saint Joseph Hospital/ University Hospital Department (DHU) Risks in Pregnancy, Paris Descartes University, Paris, France

**Keywords:** Pregnancy outcome, Epidemiology

## Abstract

We investigated the extent to which pre-pregnancy obesity mediates the association between maternal place of birth and severe pre-eclampsia in the PreCARE cohort of pregnant women in Paris (n = 9,579). Adjusted path analysis logistic regression models were used to assess the role of pre-pregnancy obesity as a mediator in the association between maternal place of birth and the development of severe pre-eclampsia. We calculated 1. adjusted odds ratios and 95% confidence intervals for the total exposure-outcome association and for the direct and indirect/obesity-mediated components 2. the indirect/obesity-mediated effect. Ninety-five (0.99%) women developed severe pre-eclampsia, 47.6% were non-European immigrants, 16.3% were born in Sub-Saharan Africa, and 12.6% were obese (BMI > = 30 kg/m^2^). Women experiencing severe pre-eclampsia were more likely to be from Sub-Saharan Africa (p = 0.023) and be obese (p = 0.048). Mothers from Sub-Saharan Africa had an increased risk of severe pre-eclampsia compared to European-born mothers (aOR 2.53, 95% CI 1.39–4.58) and the obesity-mediated indirect effect was 18% of the total risk (aOR 1.18, 95%CI 1.03–1.35). In conclusion, Sub-Saharan African immigrant women have a two-fold higher risk of developing severe pre-eclampsia as compared to European-born women, one-fifth of which is mediated by pre-pregnancy obesity. Our results quantify the potential benefit of decreasing obesity among at-risk women.

## Introduction

Pre-eclampsia, a disorder of pregnancy characterized by hypertension and proteinuria, affects 3–5% of pregnancies globally^1–3^ and is one of the leading causes of maternal and neonatal mortality and morbidity in developed countries^2,4–7^. Severe pre-eclampsia is characterized by higher blood pressures and more profound organ dysfunction than mild pre-eclampsia^8^ and constitutes the largest attributable fraction of severe morbidity due to hypertensive disorders in pregnancy^5^. Overall, the prevalence of pre-eclampsia has been declining in some European countries and Australia but continues to rise in the United States^5,9,10^. The increase in pre-eclampsia in the United States is driven specifically by higher rates of severe disease: while mild pre-eclampsia declined between 1980 and 2010, from 3.1% to 2.5%, severe pre-eclampsia increased from 0.3% to 1.4%^11^.

There are known social and medical factors associated with a differential risk of developing pre-eclampsia. Maternal origin has been linked to disparities in rates of pre-eclampsia in various settings. In Europe, immigrant women from Sub-Saharan Africa, Latin America, and the Caribbean have a higher risk of pre-eclampsia^12–14^ as have women from minority racial (Black) or ethnic (African and Turkish) groups^15,16^. In the United States, Black and Latina mothers suffer from disproportionately higher rates of pre-eclampsia as compared to white mothers^17–20^. Obesity has also been shown to be associated with an increased risk of severe pre-eclampsia^21^. Maternal obesity rates increased from 6.0% to 11.8% between 1998–2016 in France and from 17.6% to 20.5% in the USA between 2003–2009, with evidence of accelerating incidence^22–24^. Of note, immigrant and minority women have disproportionately increased rates of pre-pregnancy obesity^23,25^, suggesting that several risk factors for pre-eclampsia often co-exist.

The aforementioned evidence demonstrates strong associations between maternal origin, obesity, and disparities in the development of pre-eclampsia in pregnancy. However the relationship between these risk factors and the development of severe, life-threatening pre-eclampsia is less well established. Furthermore the precise role of obesity, a modifiable target for disease prevention and treatment, has not been well elucidated on the causal pathway^26^. We hypothesized that obesity may be a mediator in the relationship between maternal origin and the occurrence of severe life-threatening pre-eclampsia. The objective of the current investigation was to test for and quantify this mediation effect.

## Methods

### Sample

The PreCARE multicenter cohort study was designed to evaluate the association between social deprivation and perinatal outcomes and to investigate the mechanisms of social health inequalities^27^. All women registered to deliver or who delivered at one of the four participating university hospitals in Paris between October 2010-November 2011 were eligible for study inclusion (n = 10,419). The study was conducted in predominantly immigrant, low-income neighborhoods of Paris. Informed consent was obtained orally at the time of study enrolment and prior to data collection, in accordance with French law. The study was approved by the *Comité de Protection des Personnes*, (CPP Ile de France III, No. 09.341bis), and the *Commission Nationale de l’Informatique et des Libertés* (CNIL), the national French data protection agency on 19 November 2009. All methods were performed in accordance with the relevant guidelines and regulations. Women younger than age 18 (n = 54), not pregnant (n = 1), who had a termination of pregnancy before 20 weeks gestational age (n = 106), delivered elsewhere (n = 210), were lost to study follow up (n = 408), had missing study questionnaire responses (n = 26), missing provider questionnaires (n = 29), or withdrew consent at any time (n = 6) were excluded from the final study sample (n = 9579, 92% of original sample).

### Study procedures

Study participants completed a self-administered questionnaire at enrollment and included detailed information regarding their medical antenatal care, psychological and social work consultations, housing and living arrangements, sources of income, public assistance, health insurance type, nationality and, if not a citizen of the European Union, legal status in France. A second similar questionnaire was completed in the postpartum period during the obstetric hospitalization and included additional questions regarding prenatal care utilization. Research assistants and interpreters were available for those who needed assistance with completing the questionnaires or non-French speakers. Questionnaires were also available in the three most common languages among non-French speakers (English, Chinese, and Romanian). Details regarding participants’ medical history, current pregnancy, and obstetric hospitalization were collected via medical chart review prospectively by research assistants and medical providers (obstetricians and midwives). Providers also completed detailed questionnaires regarding participants’ delivery and postpartum course.

The binary outcome of severe pre-eclampsia was defined as having pre-eclampsia and one or more of the following: a systolic blood pressure >160 mmHg or diastolic blood pressure >110 mmHg, proteinuria >3.5 g/24 h, serum creatinine >100 µmol/l, urine output <20 ml/h, hemolysis, liver transaminitis >3 times upper limit of normal, thrombocytopenia <100 000/mm^3^, or gestational age <32 weeks at onset of disease. The definition of severe pre-eclampsia used in the current investigation is based on the French guidelines at the time of study protocol preparation^28^. The exposure of interest in the current analysis was maternal place of birth coded in four categories: France and Europe, North Africa, Sub-Saharan Africa, and other. Pre-pregnancy BMI was abstracted from the medical chart and was calculated by self-reported height and weight at the first prenatal visit. Pre-pregnancy body mass index (BMI) was the mediating factor in the analysis and was coded according to standard clinical categories: underweight (BMI < 18.5 kg/m^2^), normal weight (BMI 18.5–24.9 kg/m^2^), overweight (BMI 25–29.9 kg/m^2^), and obese (BMI > = 30 kg/m^2^).

Potential confounding variables were either for the causal effect of the exposure on the outcome or of the mediator on the outcome^29^ and did not include intermediate factors^30^.

Confounding factors considered were: maternal age as a continuous variable, parity as a binary variable (primiparous or multiparous), and a constructed composite maternal social deprivation variable which was defined as per a previously-validated index and included meeting any one of the following four criteria: being socially isolated, living in poor housing conditions, having no work-related household income, or having no standard health insurance^27^. Prenatal care utilization and chronic hypertension were not included as confounders in the primary analysis as they are potential mediators in the causal pathway between maternal place of birth and severe pre-eclampsia.

### Statistical analysis

Demographic, social, and medical characteristics of participants were described and differences between women who did and did not experience severe pre-eclampsia ascertained using chi-squared tests of independence or Fisher’s exact tests for categorical variables, and two sample t-tests or ANOVA for continuous variables (p < 0.05). Since there was no association between being overweight and developing severe pre-eclampsia in our sample, we did not include overweight women in our mediation analysis. Furthermore, as our aim was to isolate the effect of obesity as compared to normal weight, the path analysis was conducted on a sub-group of the entire study sample which consisted of women who were either normal weight or obese (n = 6476)^31^(31). A path analysis multivariable logistic regression model was used to decompose the total effect of maternal place of birth on severe pre-eclampsia into an indirect effect, that mediated by obesity as compared to a reference with normal weight, and a residual, so called direct, effect not mediated by obesity^31,32^. The path analysis model conceptualized a counterfactual approach which may theoretically be formulated as the response to the following question: “What would be the risk of severe pre-eclampsia associated with foreign maternal place of birth if foreign-born women had the same probability of being obese as women born in Europe?”. Two estimates of the direct and indirect effects can be made, either based on the answer to the counterfactual question above or on another possible question: “What would be the risk of severe pre-eclampsia associated with foreign maternal place of birth if European-born women had the same probability of being obese as immigrant-born women?”. The two estimates are usually very similar, and if so the direct and indirect (i.e. obesity-mediated) estimates corresponding to the first counterfactual question are reported. The indirect obesity-mediated effect of maternal place of birth on the risk of severe pre-eclampsia was calculated from the regression coefficients obtained via adjusted logistic regression models and was expressed as a percentage of the total effect.

Two sensitivity analyses were performed. The first revised the path analysis mediation model to include an additional adjustment for prenatal care utilization during the pregnancy which was coded in two categories (prenatal care visits as a proportion of what is routinely recommended by gestational age at delivery either <50% or > =50%^33,34^). We conducted this sensitivity analysis to ascertain if treating prenatal care utilization as a confounder as opposed to an intermediate variable changed the obesity-mediated association between maternal place of birth and severe pre-eclampsia significantly. Given legal limitations on the routine collection of data regarding race in France, an additional sensitivity analysis was performed to account for potential differences between maternal place of birth and self-reported ethnic origin by using the latter as the exposure of interest in the path analysis mediation model.

All analyses were performed using Stata, V.14.0 SE (Stata Corporation, College Station, Texas, USA) and add-on models developed by Buis^31^.

## Results

Ninety-five out of 9579 women (0.99%) in our sample experienced severe pre-eclampsia during the course of their pregnancies. Differences in the socio-demographic characteristics, medical history, current pregnancy, and delivery characteristics of participants with and without severe preeclampsia are presented in Tables 1 and 2. Mothers experiencing severe pre-eclampsia were more likely to be immigrants from Sub-Saharan Africa, be obese, not be living with the father of the child, suffer from overall social deprivation, have chronic hypertension, and be primiparous (p < 0.05). They were also more likely to have had less than 50% of recommended antenatal consultations, conceived via assisted reproductive technologies, have a multiple gestation, be diagnosed with a high risk pregnancy^33^, deliver preterm, and have a cesarean delivery (p < 0.05). Fetal deaths were higher among participants who developed severe pre-eclampsia (p < 0.05). Neonates born to mothers with severe pre-eclampsia were more likely to have low birth weight (p < 0.05). Selected characteristics of participants by maternal place of birth are described in Table 3. The prevalence of obesity differed significantly by maternal place of birth and was the highest in women born in Sub-Saharan Africa (Table 3).

**Table 1:** Socio-demographic characteristics and medical history of participants with and without severe preeclampsia.

	All participants	Did not experience severe preeclampsia	Experienced severe preeclampsia*	p**
	N = 9579	n = 9484 (99.01%)	n = 95 (0.99%)	
**Socio-demographic information**				
Age (years)	30.8 ± 5.4	30.8 ± 5.4	31.3 ± 5.1	0.343
Maternal place of birth (n,%)				0.023
France + Europe	4979 (52.4%)	4933 (52.5%)	46 (48.9%)	
North Africa	2106 (22.2%)	2093 (22.3%)	13 (13.8%)	
Sub-Saharan Africa	1558 (16.4%)	1533 (16.3%)	25 (26.6%)	
Other	855 (9.0%)	845 (9.0%)	10 (10.6%)	
Length of stay in France				0.256
<1 year or not living in France	660 (7.5%)	657 (7.6%)	3 (3.4%)	
>= 1 year	3889 (44.2%)	3845 (44.1%)	44 (50.0%)	
Living in France since birth	4259 (48.4%)	4218 (48.4%)	41 (46.6%)	
Language barrier (n, %)				0.836
Yes - total	192 (2.1%)	191 (2.1%)	1 (1.1%)	
Yes - partial	812 (8.7%)	803 (8.7%)	9 (9.9%)	
No	8304 (89.2%)	8,233 (89.2%)	81 (89.0%)	
Living with father of child (n, %)				<0.001
Yes	8129 (85.3%)	8060 (85.4%)	69 (72.6%)	
No	1405 (14.7%)	1379 (14.6%)	26 (27.4%)	
Education (n, %)				0.226
High school diploma	4698 (49.6%)	4645 (49.6%)	53 (55.8%)	
Beyond high school diploma	4772 (50.4%)	4730 (50.5%)	42 (44.2%)	
Overall social deprivation*** (n, %)	3211 (33.6%)	3170 (33.5%)	41 (43.2%)	0.047
**Medical history**				
Prepregnancy BMI (n, %)				
(collapsed all obese categories)				0.048
Normal weight (BMI 18.5–24.9 kg/m^2^)	5380 (59.2%)	5338 (59.3%)	42 (47.2%)	
Underweight (BMI < 18.5 kg/m^2^)	534 (5.9%)	526 (5.9%)	8 (9.0%)	
Overweight (BMI 25–29.9 kg/m^2^)	2027 (22.3%)	2006 (22.3%)	21 (23.6%)	
Obese (BMI > = 30 kg/m^2^)	1145 (12.6%)	1127 (12.5%)	18 (20.2%)	
Tobacco use (n, %)	1626 (17.1%)	1612 (17.2%)	14 (14.7%)	0.533
Chronic hypertension (n, %)	152 (1.6%)	140 (1.5%)	12 (12.6%)	<0.001
**Obstetric history**				
Parity (n, %)				<0.001
Primiparous	4103 (42.9%)	4042 (42.7%)	61 (64.2%)	
Multiparous	5467 (57.1%)	5433 (57.3%)	34 (35.8%)	
*Among multiparous participants*				
Prior cesarean delivery (n, %)	1179 (12.3%)	1169 (21.5%)	10 (29.4%)	0.593
Prior preeclampsia (n, %)	104 (1.1%)	101 (1.9%)	3 (8.8%)	0.084

*Defined as having pre-eclampsia and one or more of the following: a systolic blood pressure >160 mmHg or diastolic blood pressure >110 mmHg, proteinuria >3.5 g/24 h, serum creatinine >100 µmol/l, urine output <20 ml/h, hemolysis, liver transaminitis >3 times upper limit of normal, thrombocytopenia <100 000/mm^3^, or gestational age <32 weeks.

**Chi-squared test of independence or Fisher’s exact test for categorical variables, t-test for continuous variables.

***Any of the following: (1) socially isolated (2) poor housing conditions (3) no work-related household income (4) no standard health insurance.

**Table 2:** Current pregnancy and delivery characteristics of participants with and without severe preeclampsia.

	All participants	Did not experience severe preeclampsia	Experienced severe preeclampsia*	p**
	N = 9579	n = 9484 (99.01%)	n = 95 (0.99%)	
**Current pregnancy**				
Recommended prenatal consultations (% of recommended)^†^				0.008
<50%	273 (2.9%)	266 (2.8%)	7 (7.4%)	
>50%	9271 (97.1%)	9183 (97.2%)	88 (92.6%)	
High risk pregnancy^†^	1867 (19.6%)	1837 (19.5%)	30 (31.9%)	0.002
ART (n, %)	373 (3.9%)	362 (3.8%)	11 (11.6%)	<0.001
Multiple gestation (n, %)	296 (3.1%)	286 (3.0%)	10 (10.5%)	<0.001
Gestational diabetes (n, %)	1006 (10.6%)	993 (10.6%)	13 (13.8%)	0.308
**Delivery**				
Gestational age (n, %)				<0.001
<28 weeks	95 (1.0%)	86 (0.9%)	9 (9.5%)	
28–37 weeks	1351 (14.1%)	1294 (13.6%)	57 (60.0%)	
>37 weeks	8133 (84.9%)	8104 (85.5%)	29 (30.5%)	
Mode of delivery (n, %)				<0.001
Vaginal birth	7531 (79.7%)	7493 (80.2%)	38 (40.0%)	
Cesarean delivery - without trial of labor	819 (8.7%)	780 (8.3%)	39 (41.1%)	
Cesarean delivery - after trial of labor	1094 (11.6%)	1076 (11.5%)	18 (19.0%)	
Maternal death	0(0.0%)			n/a
**Newborn**				
Fetal death	79 (0.8%)	72 (0.8%)	7 (7.4%)	<0.001
Intra-uterine demise + stillbirth	49 (0.5%)	48 (0.5%)	1 (1.1%)	0.388
Induced abortion	30 (0.3%)	24 (0.3%)	6 (6.3%)	<0.001
Birth weight (g)				
Birth weight <10thpercentile	889 (9.4%)	866 (9.2%)	23 (26.7%)	<0.001
Birth weight <3rd percentile	343 (3.6%)	332 (3.5%)	11 (12.8%)	<0.001
Neonatal death (n, %)	15 (0.2%)	14 (0.2%)	1 (1.1%)	0.133

*Defined as having pre-eclampsia and one or more of the following: a systolic blood pressure >160 mmHg or diastolic blood pressure >110 mmHg, proteinuria >3.5 g/24 h, serum creatinine >100 µmol/l, urine output <20 ml/h, hemolysis, liver transaminitis >3 times upper limit of normal, thrombocytopenia <100 000/mm^3^, or gestational age <32 weeks.

**Chi-squared test of independence or Fisher’s exact test for categorical variables, t-test for continuous variables.

^†^As per the French Haute Autorité de Santé recommendations, 2016.

**Table 3:** Demographic and social characteristics of participants by maternal place of birth.

	France + Europe	North Africa	Sub-Saharan Africa	Other	p*
	N = 4979 (52.4%)	N = 2106 (22.2%)	N = 1558 (16.4%)	N = 855 (9.0%)	
Age (years)	30.5 ± 5.2	31.6 ± 5.6	30.9 ± 5.6	30.8 ± 5.5	<0.001
Length of stay in France					<0.001
<1 year or not living in France	76 (1.6%)	293 (16.0%)	190 (14.2%)	95 (13.1%)	
>= 1 year	552 (11.3%)	1544 (84.1%)	1144 (85.8%)	631 (86.9%)	
Living in France since birth	4250 (87.1%)	0 (0.0%)	0 (0.0%)	0 (0.0%)	
Language barrier (n, %)					<0.001
No	4801 (96.8%)	1653 (83.9%)	1261 (84.9%)	527 (64.2%)	
Yes - partial	109 (2.2%)	281 (14.3%)	201 (13.5%)	214 (26.1%)	
Yes -total	49 (1.0%)	37 (1.9%)	23 (1.6%)	80 (9.7%)	
Living with father of child (n, %)					<0.001
Yes	4421 (88.9%)	1968 (93.5%)	957 (61.6%)	750 (87.8%)	
No	555 (11.2%)	137 (6.5%)	597 (38.4%)	104 (12.2%)	
Education (n, %)					<0.001
High school diploma	1788 (36.0%)	1238 (59.3%)	1110 (72.3%)	528 (62.5%)	
Beyond high school diploma	3175 (64.0%)	849 (40.7%)	425 (27.7%)	317 (37.5%)	
Overall social deprivation** (n, %)					<0.001
Yes	1039 (20.9%)	823 (39.1%)	937 (60.1%)	377 (44.1%)	
No	3940 (79.1%)	1283 (60.9%)	621 (39.9%)	478 (55.9%)	
Prepregnancy BMI (n, %)					<0.001
Normal weight	3145 (64.9%)	1049 (52.0%)	626 (45.6%)	522 (67.3%)	
Underweight	332 (6.9%)	69 (4.0%)	57 (4.2%)	72 (9.2%)	
Overweight	835 (17.2%)	630 (31.2%)	411 (29.9%)	130 (16.8%)	
Obese	533 (11.0%)	270 (13.4%)	279 (20.3%)	52 (6.7%)	
Parity (n, %)					<0.001
Primiparous	2523 (50.7%)	763 (36.3%)	419 (26.9%)	362 (42.4%)	
Multiparous	2451 (49.3%)	1342 (63.8%)	1138 (73.1%)	492 (57.6%)	
Recommended prenatal consultations (% of recommended)^†^					0.065
<50%	131 (2.6%)	49 (2.3%)	58 (3.7%)	23 (2.7%)	
>50%	4831 (97.4%)	2050 (97.7%)	1494 (96.3%)	831 (97.3%)	

*Chi-squared test of independence or Fisher’s exact test for categorical variables, ANOVA for continuous variables.

**Any of the following: (1) socially isolated (2) poor housing conditions (3) no work-related household income (4) no standard health insurance.

^†^As per the French Haute Autorité de Santé recommendations, 2016.

Excluding missing data for severe pre-eclampsia outcome (n = 29) and maternal place of birth (n = 81).

**Figure 1:**
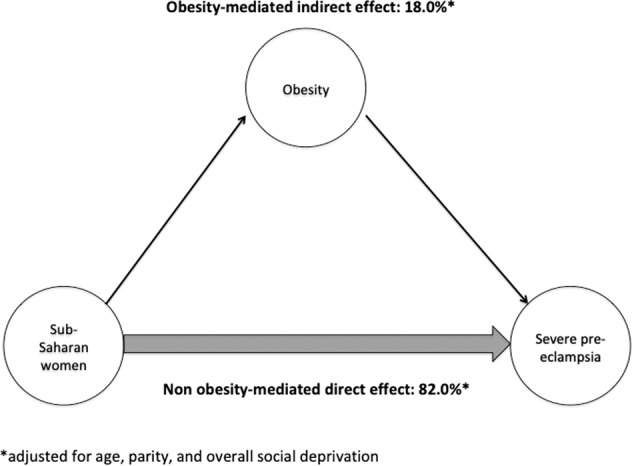
Decomposition of the total effect of Sub-Saharan maternal place of birth on the risk of severe pre-eclampsia into a direct effect and an indirect effect mediated through obesity.

There was no association between being overweight and developing severe pre-eclampsia (OR 1.33 95%CI 0.79–2.25), therefore we limited our path analysis to obese versus normal weight women only. The results of the logistic regression multivariable mediation models are presented in Table 4. Immigrant women from Sub-Saharan Africa had a higher risk of developing severe pre-eclampsia in the main analysis, total aOR 2.53, 95%CI 1.39–4.58. In the path analysis, the direct residual effect of this association was aOR 2.14, 95%CI 1.15–3.99 and the indirect effect, i.e. that mediated by obesity, was aOR 1.18, 95%CI 1.03–1.35, which corresponds to an 18% indirect obesity-mediated effect (Fig. 1).

**Table 4:** Obesity-mediated effect of maternal place of birth on severe preeclampsia*.

	Total unadjusted OR (95%CI)	Adjusted for age, parity**, and overall social deprivation***		
		Total adjusted aOR (95%CI)	Direct aOR (95%CI)	Obesity-mediated indirect aOR (95%CI)
N	6476	6473		
Maternal place of birth				
France and Europe	1.00	1.00	1.00	1.00
North Africa	0.77 (0.33–1.81)	0.80 (0.33–1.94)	0.75 (0.31–1.81)	1.07 (1.00–1.14)
Sub-Saharan Africa	2.27 (1.00–5.13)	**2.53 (1.39–4.58)**	**2.14 (1.15–3.99)**	**1.18 (1.03–1.35)**^†^
Other	1.33 (0.47–3.75)	1.34 (0.59–3.06)	1.43 (0.62–3.31)	0.94 (0.86–1.02)

*Obesity (BMI > = 30 kg/m^2^); reference: normal weight (BMI 18.5–24.9 kg/m^2^).

**Primiparous or multiparous.

***Any of the following: (1) socially isolated (2) poor housing conditions (3) no work-related household income (4) no standard health insurance.

^†^Indirect effect: 18.0%.

The results of the sensitivity analyses examining the effect of including prenatal care utilization as a control variable found a similar 18% indirect obesity-mediated (Supplementary Table 1). The findings of the sensitivity analysis examining the obesity-mediated effect of self-reported maternal origin, as opposed to maternal place of birth, on severe preeclampsia were similar to that of the principal investigation (Supplementary Table 2).

## Discussion

### Main findings

We found that women born in Sub-Saharan Africa had more than twice the risk of developing severe pre-eclampsia as compared to women born in Europe. Most notably our results suggest that almost one-fifth of this elevated risk is mediated by pre-pregnancy obesity, while accounting for potential confounders. The latter is a novel finding that provides important insights to the mechanisms by which disparities in the risk of severe life-threatening hypertensive disorders among pregnant women operate along social determinants and offers a target for prevention and treatment, i.e. maternal obesity.

### Interpretation

The findings of the current investigation are consistent with previous evidence that immigrant women in Europe from Sub-Saharan Africa or those of African ethnicity have an increased risk of developing severe pre-eclampsia^13,16^. To our knowledge, there are no prior studies evaluating the role of obesity as a mediator in the relationship between maternal origin and severe pre-eclampsia. Snowden *et al*. examined the independent and joint effects of maternal obesity and race/ethnicity on various perinatal outcomes including pre-eclampsia generally in a large retrospective cohort of all births in California in 2007 (N = 385,407)^35^. Their analysis differed critically from the present study in several ways. The exposure was maternal race, a social construct for which the implications are likely different than maternal place of birth. The outcome was not restricted to severe pre-eclampsia. Furthermore, they used a different statistical approach. Nonetheless we believe it still provides a potential comparison when attempting to understand the relationships between maternal origin, obesity, and pre-eclampsia – particularly in the absence of more congruous prior studies. In the aforementioned investigation, compared with white women, African American women had a higher risk of pre-eclampsia (aOR 1.60, 95%CI 1.48–1.74), as did obese African American women compared to obese white women (aOR 1.45, 95%CI 1.24–1.70). Our findings are broadly consistent with these results. Of interest however, they found an attenuated risk associated with increasing weight, suggesting that BMI is not the only driver of the association between maternal race and pre-eclampsia. The 18% mediation effect of obesity in our study suggests a possibly congruent finding, while additionally quantifying the effect and providing an estimate of what potential reduction in rates of severe pre-eclampsia may be expected by targeting maternal obesity. Since obesity only explains a minor, although important, part of the association between Sub-Saharan place of birth and severe pre-eclampsia, our results also indicate that other factors may be mediating this relationship given the magnitude of the direct effect (82%) which is in fact residual and unexplained. One such candidate mediator may be differential quality of care for ethnic minorities or immigrants. Indeed, data show that immigrants from Sub-Saharan Africa in France have been shown to have non-medically justifiable delays in prenatal follow up and diagnosis of pre-eclampsia^36^ and in the United Sates, differential quality of care for African American mothers who go on to develop severe morbid pregnancy outcomes has been well-documented^37,38^. Our findings therefore indicate the need not only to reduce pre-pregnancy obesity via pre-conception care, nutrition, exercise, knowledge and access, but also to improve our understanding of other modifiable drivers of severe pre-eclampsia among at-risk minority or immigrant women regardless of their BMI.

### Strengths and limitations

Our investigation had several strengths including the prospective study design and a large prevalence of non-European immigrant participants (47.6%), which allowed us to better examine health outcomes in this important subgroup of pregnant women. The high retention rate of 92% and low loss to follow up of the final analyzed sample was a significant strength of this study which limited selection bias in this social cohort study. The dataset included an exhaustive list of social variables from the participant questionnaires, most of which are not otherwise routinely collected in France. The study was conducted in relatively socially deprived neighborhoods of Paris^27,39^ and nearly 11% of participants had a language barrier, representing an important and previously under-investigated population in France. The maternal obesity prevalence (12.6%) was similar to the French national average (11.8%)^24^, allowing for generalizability in that dimension.

Our study also has limitations. The relatively small number of severe pre-eclamptic outcomes was a limitation of our statistical analysis. Pre-pregnancy BMI was calculated from self-reported weight, which may be inaccurate however more likely to underestimate obesity in our sample and thus the overall effect. Furthermore, the magnitude of this reporting bias is likely to be negligible^40^. The definition of severe pre-eclampsia used in our study is as per the French national clinical guidelines at the time of protocol development, which have since changed^28^. However this has likely not affected our main results significantly. A more complex mediation model would include factors that could account for the residual or direct effect and allow for a more nuanced understanding of the various mediators of the associations. However we believe focusing our analysis on a single mediator allowed us to not only isolate pre-pregnancy obesity as a risk factor but also to provide a statistically demonstrative baseline for future, more complex analyses. Finally, generalizability of our results to other countries must be considered carefully given obesity prevalence, health systems, and immigrant and minority health vary considerably by national context.

Immigrant women from Sub Saharan Africa have a twofold higher risk than European-born women of developing severe pre-eclampsia and one fifth of this elevated risk may be mediated by pre-pregnancy obesity, a modifiable risk factor and a potential target for prevention and treatment interventions. While our findings improve our understanding of the complex relationships between maternal origin, obesity, and severe pre-eclampsia, they also highlight the need to better understand other drivers of severe maternal outcomes among immigrant women. Future investigations should focus on better elucidating the role of other modifiable mediators, such as those pertaining to quality of care and the health care system factors in an effort to improve

## Supplementary information

Supplementary tables.

## Data Availability

Due to ethical restrictions, data will be made available on request to A. Siddiqui (ayesha.siddiqui@inserm.fr) and subject to receiving appropriate French ethical approval.
