# Supplementary material for: Maternal obesity and severe pre-eclampsia among immigrant women: a mediation analysis
Source: Sci Rep. 2020 Mar 23;10:5215. doi: 10.1038/s41598-020-62032-9 (PMC7089990; doi:10.1038/s41598-020-62032-9)
Supplement: Supplementary file 1 — Supplementary tables. [file 41598_2020_62032_MOESM1_ESM.pdf]

# Maternal obesity and severe pre-eclampsia among immigrant women: a mediation analysis

## SUPPLEMENTARY TABLES

**Authors**

Ayesha Siddiqui MD MSc[1, 2*], Catherine Deneux-Tharaux MD PhD[1], Dominique Luton MD PhD[3], Thomas Schmitz MD PhD[4], Laurent Mandelbrot MD PhD[5], Candice Estellat MD PhD[6], Elizabeth A. Howell MD MPP[7,8,9], Babak Khoshnood MD PhD[1], Nathalie Bertille PhD[1], and Elie Azria MD PhD[1,10]

**Affiliations**

[1]INSERM U1153 Obstetrical, Perinatal and Pediatric Epidemiology Research Team (EPOPé), Center for Epidemiology and Statistics Sorbonne Paris Cité, Risks in Pregnancy DHU, Paris Descartes University, Paris, France

[2]Department of Family Medicine and Community Health, Icahn School of Medicine at Mount Sinai, New York, NY, USA

[3]Department of Obstetrics and Gynecology, Beaujon-Bichat Hospital, University Hospital Department (DHU) Risks in Pregnancy, Paris Diderot University, Paris, France

[4]Department of Obstetrics and Gynecology, Robert Debré Hospital, Paris Diderot University, Paris, France

[5]Department of Obstetrics and Gynecology, Louis Mourier Hospital, University Hospital Department (DHU) Risks in Pregnancy, Paris Diderot University, Colombes, France

[6]Pitié-Salpêtrière Hospital – Charles Foix; Department of Biostatistics, Public Health and Medical Information, Clinical Research Unit, Pharmacoepidemiology Center (Céphépi), Sorbonne University; INSERM UMR-S 1136 - Pierre Louis Institute of Epidemiology and Public Health, Paris, France

[7]Department of Population Health Science and Policy, Icahn School of Medicine at Mount Sinai, New York, NY, USA

[8]Department of Obstetrics, Gynecology, and Reproductive Science, Icahn School of Medicine at Mount Sinai, New York, NY, USA

[9]Blavatnik Family Women's Health Research Institute, Icahn School of Medicine at Mount Sinai, New York, NY, USA

[10]Maternity Unit, Notre Dame de Bon Secours -Paris Saint Joseph Hospital/ University Hospital Department (DHU) Risks in Pregnancy, Paris Descartes University

***Corresponding author**

Ayesha Siddiqui, MD MSc

INSERM U115 Obstetrical, Perinatal and Pediatric Epidemiology Research Team

Center for Epidemiology and Statistics Sorbonne Paris Cité

Maternité Port-Royal 6ème étage

53 avenue de l'Observatoire

75014 Paris, France

ayesha.siddiqui@inserm.fr

**Supplementary table 1. Sensitivity analysis: Obesity*-mediated effect of maternal place of birth on severe preeclampsia - additional adjustment by prenatal care utilization**

| | | *Adjusted for: age, parity**, overall social deprivation***, and prenatal care utilization†* | | |
|---|---|---|---|---|
| | Total unadjusted OR (95%CI) | Total adjusted aOR (95%CI) | Direct aOR (95%CI) | Obesity-mediated indire[illegible] aOR (95%CI) |
| N | 6476 | 6459 | | |
| Maternal place of birth | | | | |
| France and Europe | 1.00 | 1.00 | 1.00 | 1.00 |
| North Africa | 0.77 (0.33-1.81) | 0.80 (0.36-1.78) | 0.75 (0.34-1.65) | 1.07 (1.00-1.13) |
| Sub-Saharan Africa | 2.27 (1.00-5.13) | **2.52 (1.33-4.74)** | **2.13 (1.14-3.99)** | **1.18 (1.04-1.35)††** |
| Other | 1.33 (0.47-3.75) | 1.34 (0.41-4.36) | 1.43 (0.44-4.69) | 0.94 (0.88-1.01) |

*Obesity (BMI >= 30kg/m2); reference: normal weight (BMI 18.5-24.9kg/m2)

**Primiparous or multiparous

***Any of the following: (1) socially isolated (2) poor housing conditions (3) no work-related household income (4) no standard health insurance

† >50% or <50% of recommended as per the French Haute Autorité de Santé recommendations, 2016

††Indirect effect: 18.0%

**Supplementary table 2. Sensitivity analysis: Obesity-mediated effect of self-reported maternal origin on severe preeclampsia**

| | | *Adjusted for age, parity**, and overall social deprivation**** | | |
|---|---|---|---|---|
| | Total unadjusted OR (95%CI) | Total adjusted OR (95%CI) | Direct aOR (95%CI) | Obesity-mediated indi aOR (95%CI) |
| N | 6469 | 6466 | | |
| Maternal place of birth | | | | |
| France and Europe | 1.00 | 1.00 | 1.00 | 1.00 |
| North Africa | 0.76 (0.36-1.61) | 0.87 (0.44-1.73) | 0.80 (0.41-1.57) | **1.08 (1.01-1.17)** |
| Sub-Saharan Africa | **2.24 (1.14-4.41)** | **2.75 (1.32-5.74)** | **2.34 (1.16-4.74)** | **1.18 (1.03-1.35)**†† |
| Other | 1.36 (0.57-3.24) | 1.49 (0.49-4.53) | 1.55 (0.51-4.69) | 0.96 (0.91-1.02) |

*Obesity (BMI >= 30kg/m2); reference: normal weight (BMI 18.5-24.9kg/m2)

**Primiparous or multiparous

***Any of the following: (1) socially isolated (2) poor housing conditions (3) no work-related household income (4) no standard health insurance

† >50% or <50% of recommended as per the French Haute Autorité de Santé recommendations, 2016

††indirect effect: 15.9%
